# The urgent need for integrated science to fight COVID-19 pandemic and beyond

**DOI:** 10.1186/s12967-020-02364-2

**Published:** 2020-05-19

**Authors:** Negar Moradian, Hans D. Ochs, Constantine Sedikies, Michael R. Hamblin, Carlos A. Camargo, J. Alfredo Martinez, Jacob D. Biamonte, Mohammad Abdollahi, Pedro J. Torres, Juan J. Nieto, Shuji Ogino, John F. Seymour, Ajith Abraham, Valentina Cauda, Sudhir Gupta, Seeram Ramakrishna, Frank W. Sellke, Armin Sorooshian, A. Wallace Hayes, Maria Martinez-Urbistondo, Manoj Gupta, Leila Azadbakht, Ahmad Esmaillzadeh, Roya Kelishadi, Alireza Esteghamati, Zahra Emam-Djomeh, Reza Majdzadeh, Partha Palit, Hamid Badali, Idupulapati Rao, Ali Akbar Saboury, L. Jagan Mohan Rao, Hamid Ahmadieh, Ali Montazeri, Gian Paolo Fadini, Daniel Pauly, Sabu Thomas, Ali A. Moosavi-Movahed, Asghar Aghamohammadi, Mehrdad Behmanesh, Vafa Rahimi-Movaghar, Saeid Ghavami, Roxana Mehran, Lucina Q. Uddin, Matthias Von Herrath, Bahram Mobasher, Nima Rezaei

**Affiliations:** 1 Universal Scientific Education and Research Network (USERN), , https://usern.tums.ac.ir/; 2 Research Center for Immunodeficiencies, Children’s Medical Center, Tehran University of Medical Sciences, Tehran, 14194 Iran; 3 Department of Pediatrics, University of Washington and Seattle Children’s Research Institute, Seattle, WA USA; 4 Centre for Research on Self Identity, Department of Psychology, School of Psychology, University of Southampton, Southampton, UK; 5 Wellman Center for Photomedicine, Massachusetts General Hospital, Harvard Medical School, Boston, USA; 6 Laser Research Centre, Faculty of Health Science, University of Johannesburg, Doornfontein, 2028 South Africa; 7 Department of Emergency Medicine, Massachusetts General Hospital, Harvard Medical School, Boston, MA USA; 8 University of Navarra, CIBERobn and IMDEA food, International Union of Nutritional Sciences (IUNS), Navarra, Spain; 9 International Union of Nutritional Sciences (IUNS), London, UK; 10 Skolkovo Institute of Science and Technology, Moscow, Russia; 11 Pharmaceutical Sciences Research Center (PSRC), The Institute of Pharmaceutical Sciences (TIPS), and School of Pharmacy, Tehran University of Medical Sciences, Tehran, Iran; 12 Departamento de Matemática Aplicada, Universidad de Granada, 18071 Granada, Spain; 13 Instituto de Matemáticas, Universidade de Santiago de Compostela, Santiago De Compostela, Spain; 14 Department of Epidemiology, Harvard T.H. Chan School of Public Health, Harvard University, Boston, MA USA; 15 Program in MPE Molecular Pathological Epidemiology, Department of Pathology, Brigham and Women’s Hospital, Harvard Medical School, Boston, MA USA; 16 Broad Institute of MIT and Harvard, Cambridge, MA USA; 17 The Sir Peter MacCallum Department of Oncology, University of Melbourne, Melbourne, Australia; 18 Department of Haematology, Peter MacCallum Cancer Centre and Royal Melbourne Hospital, Melbourne, Australia; 19 Machine Intelligence Research Labs, Auburn, WA USA; 20 Department of Applied Science and Technology, Politecnico di Torino Corso, Duca degli Abruzzi 24, 10129 Turin, Italy; 21 Division of Basic and Clinical Immunology, University of California Irvine, California, USA; 22 National University of Singapore, Singapore, Singapore; 23 Division of Cardiothoracic Surgery, Department of Surgery, Rhode Island Hospital, Alpert Medical School of Brown University, Providence, RI 02903 USA; 24 Department of Chemical and Environmental Engineering, University of Arizona, Tucson, AZ USA; 25 Department of Hydrology and Atmospheric Sciences, University of Arizona, Tucson, AZ USA; 26 A. Wallace Hayes, University of South, Florida College of Public Health and Institute for Integrative Toxicology, Michigan State University, East Lansing, USA; 27 Internal Medicine and Infections Service, Hospital Puerta De Hierro, Madrid, Spain; 28 Department of Mechanical Engineering, National University of Singapore, Singapore, Singapore; 29 Department of Community Nutrition, School of Nutritional Sciences and Dietetics, Tehran University of Medical Sciences, Tehran, Iran; 30 Child Growth and Development Research Center, Research Institute for Primordial Prevention of Non-communicable Disease, Isfahan University of Medical Sciences, Isfahan, Iran; 31 Endocrinology and Metabolism Research Center, Tehran University of Medical Sciences, Tehran, Tehran, Iran; 32 Department of Food Science, Engineering and Technology, College of Agriculture & Natural Resources, University of Tehran, Karaj Campus, Karaj, Iran; Transfer Phenomena Laboratory (TPL), Controlled Release Center, University of Tehran, Karaj Campus, Karaj, Iran; 33 Department of Epidemiology and Biostatistics, School of Public Health, Tehran University of Medical Sciences, Tehran, Iran; 34 Department of Pharmaceutical Sciences, Drug Discovery Research Laboratorty, Assam University, Silchar, Assam India; 35 Invasive Fungi Research Center and Department of Medical Mycology, School of Medicine, Mazandaran University of Medical Sciences, Sari, Iran; 36 Fungus Testing Laboratory, Department of Pathology and Laboratory Medicine, University of Texas Health Science Center at San Antonio, San Antonio, USA; 37 Centro Internacional de Agricultura Tropical (CIAT), Cali, Colombia; 38 Institute of Biochemistry and Biophysics, University of Tehran, Tehran, Iran; 39 Spice and Flavour Science Department, CSIR-Central Food Technological Research Institute, Mysore, India; 40 Ophthalmic Research Center, Shahid Beheshti University of Medical Sciences, Tehran, Iran; 41 Population Health Research Group, Health Metrics Research Center, Institute for Health Sciences Research, ACECR, Tehran, Iran; 42 Department of Medicine, Division of Metabolic Diseases and, Padova Hospital, University of Padova, Padua, Italy; 43 Institute for the Oceans and Fisheries, University of British Columbia, Vancouver, BC Canada; 44 School of Chemical Sciences, Mahatma Gandhi University, Kerala, 686 560 India; 45 Department of Genetics, Faculty of Biological Sciences, Tarbiat Modares University, Tehran, Iran; 46 Sina Trauma and Surgery Research Center, Tehran University of Medical Sciences, Tehran, Iran; 47 Department of Human Anatomy and Cell Science, Max Rady College of Medicine, Rady Faculty of Health Sciences, University of Manitoba, Winnipeg, MB R3E 3P4 Canada; 48 Faculty of Medicine, Katowice School of Technology, 40-555 Katowice, Poland; 49 Zena and Michael A. Weiner Cardiovascular Institute, Icahn School of Medicine at Mount Sinai and Cardiovascular Research Foundation, New York, NY USA; 50 Department of Psychology, University of Miami, Miami, USA; 51 Center for Type 1, Diabetes Research, La Jolla Institute for Immunology, La Jolla, CA USA; 52 Department of Physics and Astronomy, University of California Riverside, Riverside, CA 92521 USA

**Keywords:** Coronavirus, COVID-19, Complex problems, Collaboration, Interdisciplinarity, Public health

## Abstract

The COVID-19 pandemic has become the leading societal concern. The pandemic has shown that the public health concern is not only a medical problem, but also affects society as a whole; so, it has also become the leading scientific concern. We discuss in this treatise the importance of bringing the world’s scientists together to find effective solutions for controlling the pandemic. By applying novel research frameworks, interdisciplinary collaboration promises to manage the pandemic’s consequences and prevent recurrences of similar pandemics.

## Background

Coronavirus disease 2019 (COVID-19), the outbreak due to severe acute respiratory syndrome coronavirus 2 (SARS-CoV-2), has taken on pandemic proportions in 2020, affecting more than 1.5 million individuals in almost all countries (Date: April 8, 2020) [1]. This alarming number is still expected to be only the “tip of the iceberg”. A global approach is imperative to improve healthcare services worldwide to combat COVID-19. During this period, many scientists from around the world have been conducting projects related to this pandemic [2].

Public health is an appropriate and timely discipline for conducting interdisciplinary studies. Actions to improve public health care require new approaches including the involvement of complementary disciplines. Many disciplines—such as medicine and pharmacy, molecular and cellular biology, microbiology and biochemistry, genetics, immunology, pharmacology, nutrition, psychology, epidemiology, economics, societal needs, communication and political sciences, health and nursing care services, physics and chemistry, geography, and statistics or computational sciences for big data management—encompass research perspectives conducive to the observation, analysis, understanding, and interpretation of the health consequences of COVID-19 [3].

There are several reasons behind the imperative for interdisciplinary research and evidence-based treatment of COVID-19. One reason is the request by public health policy experts for science-based reports on the feasibility and efficiency of measures that could be helpful to decision-makers, while satisfying community needs and aspirations. Collaboration among scientists with an interdisciplinary focus can help achieve such a goal [4, 5].

## Covid-19

Coronaviruses (CoVs) are human and vertebrate pathogens [6]. They are able to infect humans, poultry, birds, insects, rodents, cats and several wild animal species, affecting not only the respiratory system, but also gastrointestinal, hepatic, cardiovascular and central nervous systems [7, 8].

The present outbreak of COVID-19, initially observed in the People’s Republic of China’s Hubei Province, has pandemically spread to every country and region of the world [9]. The World Health Organization (WHO) announced a global health emergency on January 30th, 2020, based on reports of exponentially increasing cases of COVID-19 observed in China and subsequently in other locations throughout the world [10]. This global health emergency warning came just 1 month after the first officially reported case from China [11].

Scientists, infectious disease experts and most governments around the world took immediate steps to control the disease and to carry out epidemiologic, virologic, and therapeutic investigations [12].

### The importance of interdisciplinary research

The last three decades witnessed a growing trend of collaborations among researchers with diverse backgrounds of training and education across world regions [13, 14]. The literature on the theoretical scope and benefits of such collaboration is extensive [15].

Although it is essential through multidisciplinary research to establish practical methods for large-scale disinfection treatment to inactivate SARS-CoV-2 in different environmental settings in order to reduce the risk of infection, this does not stop the pandemic. Unlike multidisciplinary research, where researchers from disparate fields work either separately or in collaboration, interdisciplinary research refers to teams with varying specialties unifying to achieve an overall objective [13, 16, 17].

In this context, Rosenfield [18] took this topic one step further by introducing a three-tiered structure for conceptualizing the mechanism of collaboration among different disciplines: (i) in *multidisciplinarity*, researchers work in parallel or sequentially from a disciplinary-specific base to address a common problem; (ii) in *Interdisciplinarity*, researchers work jointly, but still from a disciplinary-specific base to address the common problem; (iii) finally, in *transdisciplinarity,* researchers work jointly, using a shared conceptual framework drawing together disciplinary-specific theories, concepts, and approaches to address a common problem [18].

One example of transdisciplinary science relevant to the problem of COVID-19 is the integrative science of microbiology, molecular pathology (including immunology), and epidemiology [19]. This science has synthesized outcomes from analytical methods of microbiology (including virology) at laboratory level, together with statistical analytical frameworks of epidemiology, with data on human populations [19].

However, there are difficulties with sufficient contact or communication within professions. Lack of confidence, lack of expertise, complexities of healthcare, the confusing nature of healthcare environments, and lack of organization and standardization can become major obstacles to successful communication [20, 21].

Now, more than ever, we are in need of widespread scientific collaboration [22, 23]. COVID-19 is a medical problem with immense societal consequences. The world’s scientists need to come together to find the proper solution for controlling this pandemic event, manage its consequences, and prevent future recurrences of similar pandemics. We provide examples below on how this can be achieved.

### Mathematics and computer science

Mathematical and epidemiological simulation plays a pivotal role in predicting, anticipating, and controlling present and future epidemics. To better understand and model the dynamics of a specific infection, researchers need to consider the influence of many variables ranging from micro-host–pathogen interactions to host-to-host encounters, and the prevailing cultural, social, economic, and local customs worldwide [24].

In order to have an accurate prediction in a mathematical model, it is imperative to obtain a precise estimation of the involved parameters. Data fitting is the process of adjusting models to data and analyzing the accuracy of the fit. For reliable parameter estimation, it is critical to implement an accurate and transparent protocol for counting the number of infected, deceased, and recovered cases and for unifying different protocols at the international level [25, 26].

Dynamic simulation, game theory, and spatial modeling play an increasing role in understanding the biology of complex systems. These methods can also illustrate the dynamics of the relationship between pathogens and their host [27].

Two mathematical models applicable to a pandemic like COVID-19 are stochastic and SIR (susceptible-infected-recovered). Stochastic modeling entails all mathematical models with the random variable in the function assignment. That is, stochastic modeling uses random assignment for forecasting functions, which is helpful in the early stages of virus propagation [28]. The SIR model has a reasonable predictive power applied to situations where individuals infect each other directly [29]. However, in recent years spatial structures, and in particular network theory (as a model for road networks or flight connections) and metapopulation, have proved relevant in understanding the complexity of virus propagation [30, 31].

Worldwide, public health agencies use these frameworks coupled with information technologies such as bioinformatics, big data analytics, machine learning, artificial learning, sensors, and image recognition to assess and establish strategies for ever-emerging infectious diseases and to respond to outbreaks [27, 32–34].

### Physics, chemistry and engineering sciences

In epidemics of highly infectious diseases, the entire community needs to participate in containing the spread of the disease. Healthcare workers (HCWs), however, have to be especially careful because they are at significantly higher risk of infection than the general population [35]. Contact precautions by means of personal protective equipment (PPE) are the major strategy to reduce the risk. However, there is uncertainty about which type of PPE protects best, what is the optimal way to don and remove it, and how to ensure that all HCWs use PPE as instructed [36, 37].

Physicists, chemists and engineers play a key role in designing and advancing PPE structure and function to inhibit COVID-19 spread throughout the communities [38]. They help in implementing the manufacturing, production pipelines, logistics, and commercialization of PPE, along with disposable gloves, sanitizing gels, and respirators. In such an emergency context, the contribution of Universities in Engineering and Material Sciences can engage in synergy with companies that produce textiles, anti-wet coatings, ventilators and respirators, and can attempt to reconvert their materials and pipelines to products useful for combatting the pandemic. For example, material science and mechanical engineering can help to perform validation tests of newly produced PPE, and assist in the preparation of documents for approval [39–41]. Furthermore, constructive interaction between certification and regulatory entities as well as local, regional, and national governments can minimize the time to regulatory approval and delivery to the market of newly produced PPE. For example, the standard catalog UNI decided to give free access to their publications related to medical device and face mask regulations in a move to support interventions against COVID-19. The national governments allocate funds to companies to help them recover or convert their production to medical devices. Moreover, private and non-profit organizations finance small and medium enterprises (SMEs) and universities to promote the transfer of research results to markets for improving the safety of community health workers (CHWs) and, more in general, for tackling the immediate social, health and education emergencies.

In addition to PPE production, physics-based techniques play a substantial role in structural biology. The majority of biological macromolecular structures are obtained by X-ray crystallography. The development of physical technologies spurred the first forays into rational drug design, in which scientists study the structure and function of molecules in order to determine which drugs might bind to their targets, and in case of viruses like SARS-CoV-2, to prevent them from replicating [42].

### Biological sciences

Biological science provide a deeper understanding of complex pathogen-host interactions by using remarkable innovations in molecular biology and computational biology, developed during the first decade of the 21st century [43, 44]. We expect that the modern tools of molecular biology will provide insight beyond merely defining causative agents for newly discovered infectious diseases, but will also address the evolution of the COVID-19 pathogen, the persistence of infectious cycles in nature, the analysis of the causes of the pandemic, and the susceptibility mechanisms of specific host groups. This knowledge will contribute to the creation of DNA and RNA banking to study pathogenic factors encoding genes [45, 46].

The control of COVID-19 largely depends on two factors. The first is to design early diagnostic tools to estimate more accurately the extent of the pandemic and provide effective vaccines. Testing kits are an amalgamation of research based on engineering and biomedical sciences. They are designed to exhibit high accuracy and rapid analysis to minimize the community spread of the disease [47]. As for the second factor, in order to arrive at a proper vaccine, scientists must identify viral epitopes that might be used as therapeutic targets and that not only activate the immune systems but also recognize the virus as a target [48].

This virus also has arisen various hypotheses among scientists who are eager to demonstrate novel disease mechanism. According to one hypothesis proposed by Jean-Laurent Casanova of Rockefeller University, immunosenescence contributes, at least in part, to the severe disease and high mortality of the elderly. However, equally important is to explain the fatal disease occasionally observed in the young population. For example, there may be an underlying inborn error of immunity associated with severe COVID-19 that affects younger individuals.

### Medical sciences

Community health workers (CHWs) are expected to be prepared for emerging epidemics, and to promote pandemic preparedness by engaging with community-level educators and mobilizers, contributing to surveillance systems, and filling the gaps in healthcare services [49, 50].

It appears that nutritional and metabolic status play as independent roles in the evolution and clinical complications observed in COVID-19 patients. Scientists are looking for food regimens that can enhance the immune system. They suggest that bioactive compounds derived from functional foods such as *Echinacea*, Curcumin, or quercetin, due to their antioxidant and anti-inflammatory features, can strengthen the immune system. These compounds are thought to enhance the vaccine and drug effectiveness in affected individuals or even help to prevent infections [51–54]. Some researchers have demonstrated the antiviral activities of functional compounds such as bioactive peptides, lipids, polysaccharides [55–57]; however, the mechanisms that underlie their action are not well understood.

Individuals suffering from diabetes or cardiovascular diseases have more severe complications and higher mortality rate than healthy metabolically controlled counterparts, where the inflammatory condition may be associated with infection triggering factors [7, 58–61]. Genetic predisposition is also expected in some cases with severe COVID-19 phenotypes [62].

Medical staff members strive to improve the health of these patients in order to achieve better population immunity against infectious diseases epidemics and pandemics like COVID-19. This goal can be achieved by increasing public access to health care services.

### Social and economical sciences

The emergence of disease and the spread of epidemics depend on individuals’ behavior, the social structure in which they are embedded, and the political and civic environment that make particular social outcomes more likely for some than for others [63, 64]. When quarantine laws and social encounter restrictions (e.g., social distancing) are put in effect [65, 66], social scientists must be aware of the psychological, mental health, and economic aspects of these decisions. Cognitive science, brain and mind science, coupled with social and behavioral sciences, contribute insights into anxiety and loneliness associated with mental disorders under pandemic measures [67–69].

COVID-19 has immense socio-economic implications. With the rapid and comprehensive increase in international travel and trade, pandemic incidents will cause economic shock waves that go far beyond mainstream health sectors and spread beyond the pathogen’s original geographic range [70]. For example, analysts forecast that the pandemics will result in an estimated annual economic decline of 2–3% of global gross domestic product (GDP) in the coming year [71]. Economic scientists can promote the necessity for businesses to become pandemic-resilient by investing in strategic, operational, and financial resilience, and have suggested that these steps are a duty of care to society and economies.

A key component to the eradication of COVID-19 and lessening its impact on global health is inter-governmental cooperation. A coordinated effort is required so that all potential factors are taken into consideration to reduce the spread of the virus and prevent similar pandemics in the future.

## Conclusion

During the last few months of the COVID-19 pandemic, while political leaders have locked the borders of their countries, we witnessed scientists tearing down theirs, creating a global collaboration unlike any in history [22]. Never before, have so many experts in so many countries focused simultaneously on a single topic, COVID-19, and with such urgency and resolve. Nearly all other research has ground to a halt.

The role and collaboration of International Organizations related to Health such as WHO, Food and Agriculture Organization (FAO), International Union of Nutritional Science (IUNS), and Non-Governmental Organizations (NGOs) as well as international consortia such as Universal Scientific Education and Research Network (USERN) and national and international academies, have been recognized as crucial for an integrated knowledge of the novel coronavirus and impacting the effective management of COVID-19 around the planet.

## Data Availability

Not applicable.
